# Distinct population of highly malignant cells in a head and neck squamous cell carcinoma cell line established by xenograft model

**DOI:** 10.1186/1423-0127-16-100

**Published:** 2009-11-16

**Authors:** Chi-Yuan Chen, Shih-Hwa Chiou, Chih-Yang Huang, Chia-Ing Jan, Shu-Chun Lin, Ming-Long Tsai, Jeng-Fan Lo

**Affiliations:** 1 Institute of Oral Biology, National Yang Ming University, Taipei, Taiwan; 2 Department of Medical Research and Education, Taipei Veterans General Hospital, Taipei, Taiwan; 3 Graduate Institute of Chinese Medical Science and Institute of Medical Science, China Medical University, Taichung, Taiwan; 4 Institute of Basic Medical Science, China Medical University, Taichung, Taiwan; 5 Department of Health and Nutrition Biotechnology, Asia University, Taichung, Taiwan; 6 Department of Pathology, China Medical University and Hospital, Taichung, Taiwan

## Abstract

The progression and metastasis of solid tumors, including head and neck squamous cell carcinoma (HNSCC), have been related to the behavior of a small subpopulation of cancer stem cells. Here, we have established a highly malignant HNSCC cell line, SASVO3, from primary tumors using three sequential rounds of xenotransplantation. SASVO3 possesses enhanced tumorigenic ability both *in vitro *and *in vivo*. Moreover, SASVO3 exhibits properties of cancer stem cells, including that increased the abilities of sphere-forming, the number of side population cells, the potential of transplanted tumor growth and elevated expression of the stem cell marker Bmi1. Injection of SASVO3 into the tail vein of nude mice resulted in lung metastases. These results are consistent with the postulate that the malignant and/or metastasis potential of HNSCC cells may reside in a stem-like subpopulation.

## Introduction

Solid tumors are known to be composed of a heterogeneous population of cells, and it has been possible to observe that certain cell residing inside tumors have stem cell like properties including tumor growth (self-renewal), heterogeneity (differentiation) and growth in tissue environments different from the original one (metastasis) [1,2]. The identification of this small subpopulation of highly tumorigenic cancer cells in solid human tumors has led to the development of the cancer stem cell theory of tumorigenesis, which postulates that only a specific rare subpopulation of cancer cells has the ability to sustain cancer growth, and the remaining cancer cells have only limited growth potential or perhaps no growth potential at all [3]. Cancer stem cells possess three fundamental characterizations [4]. First, they have to self-renew, allowing the maintenance of the original stem cell population. Second, cancer stem cells must be able to differentiate into multiple types of mature cells. Third, the total number of stem cells is strictly regulated via both extrinsic and intrinsic mechanisms. Based on these characteristics, four key methodologies were developed to identify the cancer stem cell subpopulation [3]: first, cancer stem cell were enriched by sphere assay to form tumorspheres, in culture conditions that promote maintenance of cancer stem cells; second, Hoechst 33342 staining used to identify the side population cell that enriches for cancer stem cell; third, the small population of cancer stem cell can be serially transplanted through multiple generations; fourth, only a small portion of the cancer cells within a tumor have tumorigenic potential when transplanted into immunodeficient mice.

Experimental evidence for the existence of cancer stem cells has been reported for several tumor types, including glioma, breast, colon, prostate and HNSCC [2,5-7]. A subpopulation of cells with cancer stem cell like properties also appear to persist in cell lines derived from a wide range of malignancies[1,8-14], including cell lines derived from HNSCC [12,15]. Recent studies have shown that the initiation, progression, recurrence and metastasis of HNSCC may involve a small population of cancer stem cells [16-18]. In addition, cancer stem cells have been suspected to be the underlying reason for the incurable and relapsing nature of many metastatic tumors. Development of new therapies that target these cancer stem cells may significantly improve the clinical treatment of cancer. Therefore, it is of importance to identify, within tumors, the subpopulation of cells that display cancer stem cell properties.

We have previously reported that a small population of HNSCC cells displaying the characteristics of cancer stem cells was enriched by sphere formation [17]. In addition, the expression of the stem-related markers in cancer stem cells has been shown to negatively correlate with the survival prognosis of HNSCC patients. However, the *in vitro *tumor sphere method for the enrichment and isolation of cancer stem cells may not imitate well the *in vivo *situation, since maintaining a small population of cancer stem cells in a cancer is known to require a special microenvironment [19,20]. So far, the serial xenotransplantation method is generally considered to be the gold standard for the isolation of the small population of highly malignant cells from a solid tumor [3]. Here, we postulated that serial xenotransplantation of a cancer cell line in nude mice will reduce the number of non-malignant clones present that may have been selected during the establishment of this cell line. In this study, we employed a xenotransplatation mouse model to investigate the malignant heterogeneity of the HNSCC cells.

## Materials and methods

### Cell Culture

The human tongue cancer cell line SAS, a high-grade tumorigenic HNSCC cell line, was obtained from the Japanese Collection of Research Bioresources (Tokyo, Japan). 293FT, kindly provided by Dr. W.-Y. Hu and was used for packaging of the viral particles. Both SAS and 293FT cells were cultured in Dulbecco's modified Eagle's medium (DMEM) containing 10% fetal bovine serum, 1% L-glutamine, 0.5 mM sodium pyruvate, 1.2 g/L sodium bicarbonate, and 2.5 mM L-glutamine.

Cultivation of the cells to form tumor spheres has been described previously [17]. In brief, the cells were cultured in tumor sphere medium consisting of serum-free DMEM/F-12, N2 supplement, 10 ng/mL human recombinant bFGF, and 10 ng/mL EGF until sphere formation was observed at about 4 weeks. All culture medium, chemical compounds, and fetal bovine were purchased from Life Technologies (Grand Island, NY).

### Plasmids and Transfection, preparation of lentivirus virus particles and infection

The pLV-EF1α-GFP lentivirus that contains the green fluorescent protein (GFP) expression cassette was purchased from Biosettia Inc. (Biosettia, San Diego, CA). pCMVD8.2 and pMD.G, which expresses GAG-POL and the vesicular stomatitis virus envelope respectively, were provided by the consortium (Academia Sinica, Taipei, Taiwan). Isolation of all plasmid DNAs was carried out using a HiSpeed plasmid purification kit (Q-Biogene, Carlsbad, CA, USA) and the protocol provided by the manufacturer.

Transfection of plasmid DNA into the cells was generally done using Lipofectamine 2000 (Invitrogen, Carlsbad, CA) and the protocol provided by the manufacturer. The efficiency of transfection was determined by microscopic examination of the green fluorescence produced by the pLV-EF1α-GFP vector.

The lentiviruses were generated by cotransfecting 293FT with lentiviral vector and packaging plasmids at 37°C for 48 h. After transfection, the viral supernatant was harvested by spinning at 2000 rpm for 10 min at 4°C. The SAS cells were inoculated with 100 μL of viral stock with MEM medium, 10% FBS and 6 μg/μL polybrene. The infected cells were incubated for 2 h at 37°C, washed twice, and then cultured in complete medium until cells were harvested. The recombinant cells were separated using a FACS Calibur apparatus (Becton Dickinson) to sort out the population of GFP expressing cells from the non-transfected cells.

### Antibodies and Western blotting

The antibodies used in this study include anti-Bmi1 (Cell Signaling, Temecula, CA, USA), anti-β-Catenin (BD Biosciences), anti-p14 (Santa Cruze Biotechnology, Inc. Santa Cruz, CA, USA), anti-p21 (Santa Cruze Biotechnology), anti-phosphorylated AKT (Ser473)(Cell Signaling, Temecula, CA, USA), anti-AKT (Santa Cruze Biotechnology), and anti-GAPDH (Chemicon, Temecula, CA, USA).

For Western blot, the cell pellets were homogenized in 200 μL lysis buffer (50 mM HEPES, pH 7.5, 150 mM NaCl, 10% glycerol, 1.5 mM MgCl_2_, 1% Triton X-100) and incubated on ice for 20 min. After centrifugation at 13,000 *g *for 30 min at 4°C, the supernatant containing the protein extracts was collected. Protein concentrations were determined using the Bio-Rad protein assay. Western blot analyses were performed as described previously [21].

### Establishment of Xenograft-derived SAS cell lines

The SAS cells were infected with pLV-EF1α-GFP lentivirus and GFP-expressing cells were collected by sorting using a flow cytometer. All the animal practices in this study were in accordance with the institutional animal welfare guideline of Taipei VGH or National Yang-Ming University, Taiwan. To establish the first generation xenograft-derived SAS cell line, the GFP-expressing SAS cells (designated as SAS-GFP in this study) were injected subcutaneously into the dorsal flank of 6-week-old nude BALB/c nu/nu mice. *In vivo *GFP imaging of the tumor was carried out and each tumor's size measured as described previously [21]. The solid tissue from the tumor was then mechanically and enzymatically disaggregated into a single-cell suspension, and this was designated SASVO1. A second generation xenograft-derived SAS cell line was then established using the same protocol as above, this time using cells from the tumor produced by the SASVO1 cells; this single cell suspension was designated SASVO2. Finally, a third generation xenograft-derived SAS cell line was established from the tumor produced by SASVO2 cells, and this was designated SASVO3.

### Assays of proliferation, migration, invasion, and anchorage-independent growth

An MTT assay kit (Sigma-Aldrich) was used to analyze the cell proliferation. Specifically, 1 × 10^3 ^cells were seeded in each well of a 24-well plate, and then 10 μL of MTT solution was added to the cells, which were then incubated at 37°C for 3 hours. The supernatant was removed, and 200 μL of DMSO were added directly to the cells. The MTT color reaction was analyzed using a microplate reader set at *A*560 nm.

For the migration assay, 2 × 10^5 ^cells in a medium with low serum (0.5% FBS) were plated in the top chamber of a Transwell (Corning, Acton, MA) with a porous transparent polyethylene terephthalate membrane (8.0 μm pore size). Medium supplemented with high serum (15% FBS) was placed in the lower chamber and used as a chemoattractant. The cells were incubated for 24 h at 37°C and cotton swabs were used to remove cells that did not migrate through the pores. The cells on the lower surface of the membrane were stained with Hoechst 33258 (Sigma-Aldrich) and fluorescent cells were identified at a magnification of 100× using a fluorescence microscope (Carl Zeiss, Oberkochen, Germany). The number of fluorescent cells in a total of five randomly selected fields was counted.

Invasion assays were done in 24-well Transwell units with an 8.0 μm porous transparent polyethylene terephthalate membrane. Cells (1 × 10^5 ^per well) were added to upper chambers (filter coated with 1 mg/mL Matrigel) in 100 μL of the low serum medium. The lower chambers were filled with 500 μL high serum medium. After 24 h incubation at 37°C, cells that remained in the Matrigel or attached to the upper side of the filter were removed with cotton swabs. Cells that had migrated through the membrane to the lower surface were stained with Hoechst 33258 and counted in five different fields under a fluorescence microscope.

For anchorage-independent growth, 5 × 10^4 ^cells were mixed with 2 mL of MEM containing 0.35% agarose (Sigma-Aldrich) and 0.5% FBS, and poured onto 60-mm plastic culture dishes pre-solidified with 2 mL MEM containing 0.7% agarose and 15% FBS. The plates were incubated at 37°C for 3 weeks and then stained with 0.5% crystal violet. The number of colonies with a diameter ≥ 100 μM was scored. Triplicate samples were performed for each experiment.

### Analysis of the side population that excludes Hoechst 33342 dye

The cells were detached from the dishes and suspended at 1 × 10^6 ^cells/ml in Hanks' balanced salt solution (HBSS, Invitrogen) supplemented with 2% fetal calf serum and 10 mM HEPES (Invitrogen). These cells were then incubated at 37°C for 90 minutes with 10 g/mL Hoechst 33342 (Sigma Chemical, St Louis, MO), either alone or in the presence of 50 g/mL verapamil (Sigma Chemical). After incubation, the stained cells were collected by centrifugation, washed, and resuspended in ice-cold HBSS. Analysis of Hoechst 33342 efflux was performed using a FACSc Calibur apparatus (Becton Dickinson, Milan, Italy) with incident 350 nm ultraviolet light. The resulting fluorescence was measured at two wavelengths using 424/44 BP and 675 LP filters that allow detection of Hoechst blue and red, respectively.

### In vivo tumor growth and metastasis

The tumor cells were subcutaneously injected into the flank of six-week-old BALB/c nu/nu mice, and examined every 5 to 7 days for tumor appearance. Tumor growth was measured twice weekly until 28 days after inoculation by determining the tumor volumes using the formula (Length × Width^2^)/2. For the detection of metastasis, 5 × 10^5 ^tumor cells were injected into tail vein and the mice were sacrificed after 90 days to detect tumor colonization in the lung. *In vivo *GFP imaging of tumors was carried out and they were then measured as described in the section "Establishment of xenograft SAS cell lines".

### Immunohistochemistry

Immunohistochemistry was performed as described previously [21]. In brief, tumor specimens were fixed in formalin and embedded in paraffin before sectioning, or kept frozen in optimal cutting temperature (OCT) embedding media. Seven-micron sections were fixed in ice-cold acetone for 5 min, and air-dried. The sections were deparaffinized twice in xylene for total of 10 min, rinsed in alcohol and then alcohol/water mixtures for 5 min with each solution, and then treated with 3% hydrogen peroxide to block endogenous peroxidase. After rinsing in distilled water, antigen retrieval was done by boiling in 50 ml of 1× Trilogy for 30 min. Then, after cooling, the sections were rinsed in phosphate-buffered saline (pH 7.4) for another 5 min. The tissue sections were incubated overnight with primary antibody at 4°C and then incubated with the secondary antibody for 1 hour at room temperature. Color was developed with AEC substrate chromogen (Dako Corporation, USA) for 10 min at room temperature. The same sections were also then counterstained with 1% hematoxylin according to the manufacturer's suggested protocol (Zymed Laboratories), dehydrated, and cover slipped with Histomount (Sigma Chemical Co., USA). The sections were examined under a light microscope.

### Statistics

An unpaired *t*-test was used for the statistical analysis. The results were considered to be statistically different when the *p-*value was < 0.05.

## Results

### Xenograft-derived SAS cells display increased tumor growth in nude mice

**Figure 1:**
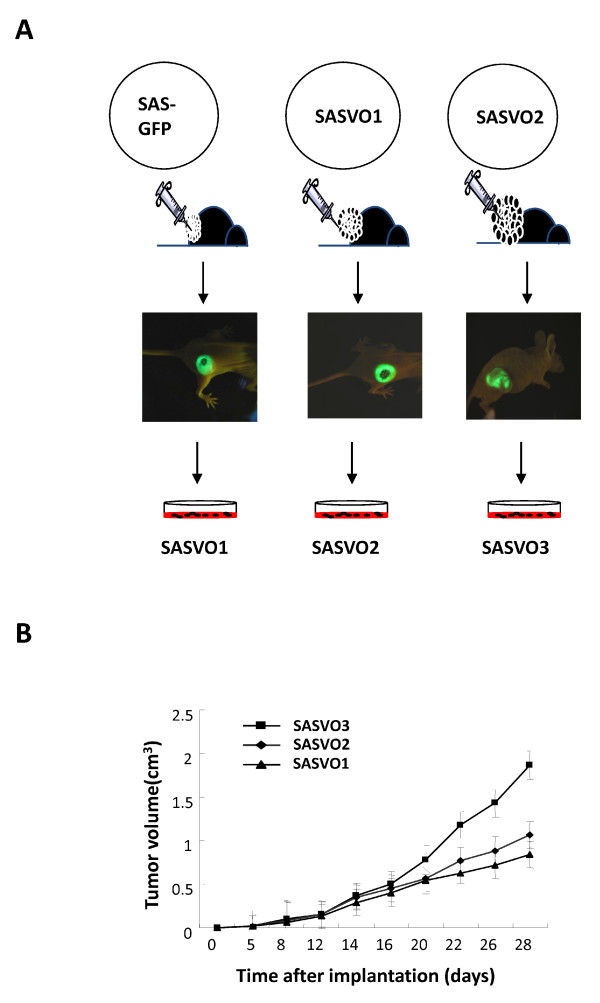
**Establishing HNSCC cell lines with increased malignant potential by the xenograft model**. (A) Experimental strategies for the generation of HNSCC cell lines with increased malignant potential. The solid tissue from the tumors produced by implantation of SAS-GFP, SASVO1 and SASVO2 cells was mechanically and enzymatically disaggregated into single-cell suspension, and designated as SASVO1, SASVO2 and SASVO3, respectively. *In vivo *GFP imaging of the tumors was carried out and the tumors were measured using an illuminating device. Pictures shown are from mice at 28 day post-injection. (B) Tumor growth of the xenograft-derived SAS cells in the nude mice. Tumors volumes were determined twice weekly. Data shown are the means ± SD.

To test the hypothesis that serial xenotransplantation of a cancer cell line in nude mice may enrich the malignant subpopulation, tumor forming SAS-GFP cells were injected subcutaneously into the dorsal flank of 6-week-old nude BALB/c nu/nu mice, and three xenograft-derived SAS cell lines, namely SASVO1, SASVO2 and SASVO3, were established from the xenograft tumors (Figure 1A). It was noted during the establishment of these xenograft-derived cell lines that the tumors generally became palpable at 5 to 7 days after inoculation, and that the expansion in size of the tumor produced by SASVO3 was faster than those of either SASVO1 or SASVO2 (Figure 1B). This would seem to indicate that the successive implantation of SAS cells in nude mice increased the ability of the tumor to grow.

### Xenograft-derived SAS cells exhibit increased capacity for proliferation, migration, invasion and anchorage-independent growth in vitro

**Figure 2:**
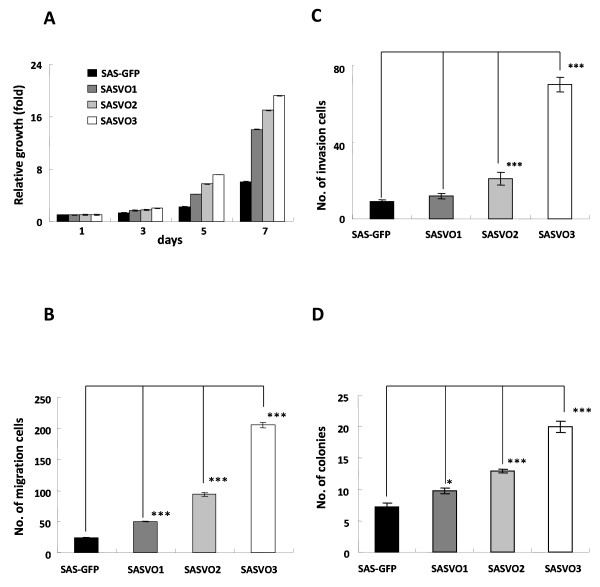
**Capacity of the xenograft-derived SAS cells for proliferation, migration, invasion and anchorage-independent growth *in vitro***. The SAS-GFP, SASVO1, SASVO2 and SASVO3 cells were assayed for (A) proliferation capacity, (B) Transwell migration ability, (C) Matrigel invasion ability and (D) anchorage independent growth as described in the Materials and Methods. Data shown are the means ± SD from three independent experiments. *, indicates *p *< 0.05; ***, indicates *p *< 0.001 when analyzed with unpaired *t*-test.

To assess the malignancy of the xenograft-derived SAS cell lines *in vitro*, the ability of these cells to proliferate, migrate, invade and to perform anchorage-independent growth was examined *in vitro*. As shown in Figure 2, the SASVO3 cells displayed the highest capacity for proliferation, migration, invasion and anchorage-independent growth and this was followed by SASVO2, then SASVO1, and finally the initial SAS-GFP cells. These *in vitro *results are consistent with the increase in tumor growth observed for the xenograft-derived SAS cells. The results indicate that successive rounds of tumor formation in mice would seem to have enriched for a subpopulation of cells with higher ability for proliferation, migration, invasion and anchorage-independent growth.

### Enrichment of cancer stem cells in SASVO3

**Figure 3:**
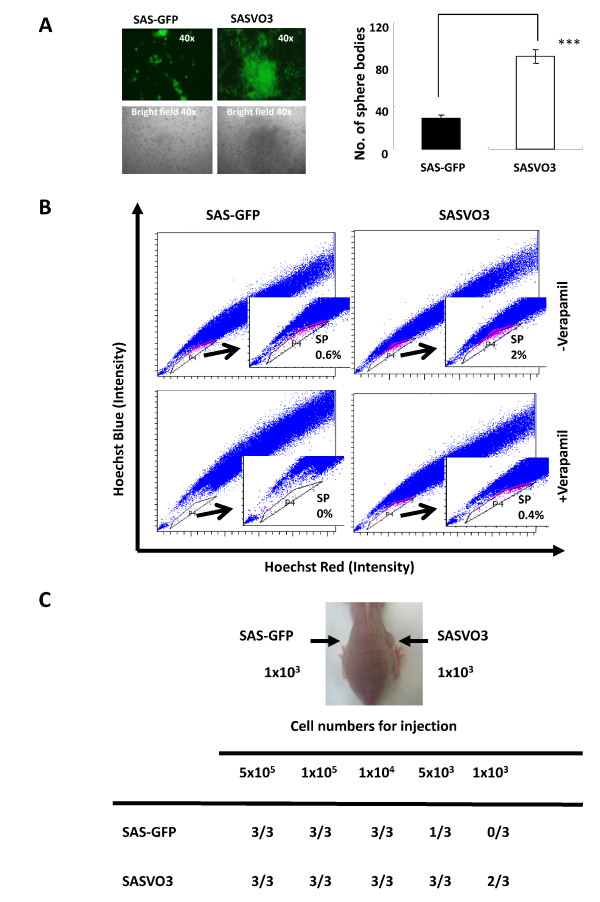
**Identification of cancer stem cells in SASVO3**. (A) Analysis of the tumor spheres generated by SASVO3 and SAS-GFP. The sizes (*left panel*) and numbers (*right panel*) of the tumor spheres formed by SAS-GFP and SASVO3 cells were analyzed by the sphere culture method as described in the Materials and Methods. Data shown are the means ± SD from three independent experiments. ***, indicates *p *< 0.001 when analyzed with unpaired *t*-test. (B) The flow cytometric profiles of side population (SP) cells that exclude Hoechst 33342 dye in SAS-GFP (*left*) and SASVO3 (*right)*. The SP cells profiles in the presence of 50 g/mL verapmil are depicted at the bottom. The area containing SP cells is marked and shown as percentage of the total cells in the inset. The result is from one of three similar experiments. (C) Tumor-initiating ability of SAS-GFP and SASVO3 in the nude mice xenograft transplant model. The picture shown on the upper panel is from mice injected with 1 × 10^3 ^cells for 28 days.

We have previously demonstrated that a subpopulation of HNSCC cells display the characteristics of cancer stem cells [17]. To address whether the xenograft-derived SASVO3 cells may be enriched for such cancer stem cells, the ability of SASVO3 cells to form tumor spheres was examined. As shown in Figure 3A, the spheres were formed more efficiently by SASVO3 cells (*right panel*), and the size of the tumor spheres formed by SASVO3 cells was about 3-fold larger than the ones formed by SAS-GFP cells (*left panel*). To further evaluate whether SASVO3 cells indeed are enriched for cancer stem cells, the side population (SP) cells that contain the cancer stem cells and possesses the ability to efflux Hoechst 33342 dye was examined [11]. As shown in Figure 3B*upper panel*, around 2% of Hoechst Low SP cells were detected among the SASVO3 cells, whereas only 0.6% of SP cells were detected among the SAS-GFP cells. Pre-incubation with verapamil (a calcium ion channel antagonist) for 30 min reduced the percentage of SP cells to 0.4% among the SASVO3 cells, but effectively eliminated all of the SP cells among the SAS-GFP cells (Figure 3B, *bottom panel*). Finally, the tumorigenic potential of the new cell line was evaluated in terms of *in vivo *tumor formation. As shown in Figure 3C, while a subcutaneous injection of 1 × 10^3 ^SAS-GFP cells produced no tumors in three mice, injection of 5 × 10^3 ^SAS-GFP cells produced a tumor in one out of three mice at 28 days. In contrast, an injection of 1 × 10^3 ^SASVO3 cells was able to generate tumors in two out of three mice, indicating that SASVO3 cells were enriched for cancer stem cells by at least 5-fold. Taken together, these results demonstrated that SASVO3 is greatly enriched in cancer stem cells.

### SASVO3 cells display an increased expression of Bmi1 and pAKT

**Figure 4:**
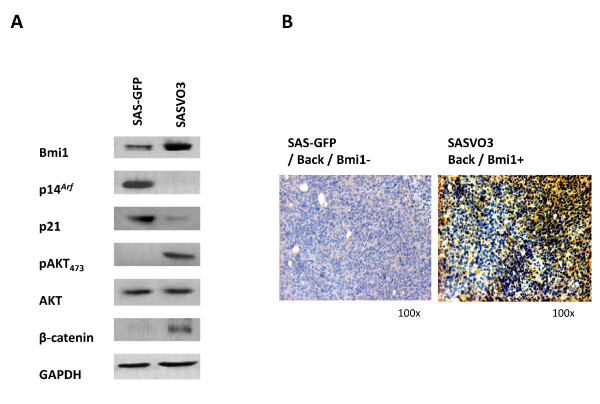
**Expression of genes unique to cancer stem cells and/or malignant cells**. (A) Cell extracts from SAS-GFP and SASVO3 were analyzed for the expression of Bmi1, p14^*Arf*^, p21, pAKT, AKT and β-catenin by Western blotting. GAPDH served as a loading control, was reproducibly similar in all experiments. Results shown are from one of three similar experiments. (B) The expression of Bmi1 protein in mouse xenografts tissue from the dorsal tumor area was detected by IHC.

To address whether the cancer stem cells enriched within SASVO3 may express molecules that are unique to stem cells and/or malignant cells, the expression levels of Bmi1, pAKT, CD133, CD44, Nanog, Oct-4, p21 and p14^*INK*4*A*/*ARF *^were determined. CD133, CD44, Nanog, and Oct-4 are known to be the common markers of cancer stem cell for HNSCC [17,18]. Bmi1 was chosen in this study because it has been implicated in the control of stem cells across multiple tissues, particularly as a positive regulator in the self renewal of hematopoietic and neuronal stem cells. It has also been implicated in the tumorigenesis of several cancers including leukemia [22,23], lung cancer [24], breast cancer [25], colorectal cancer [26] and HNSCC [27,28]. The oncogenic function of Bmi1 is mainly believed to be associated with its repressive effect on the *Ink4a/Arf *tumor suppressor locus and p53/p21 pathway, both of which plays critical roles in the onset of cellular senescence in many different types of human somatic cells [29,30]. Therefore, Bmi1 may be associated with tumorigenesis via dysregulation of multiple growth regulatory pathways. AKT was selected in this study because AKT activity is constitutively high in many cancers [31-33] and a recent study has suggested that Bmi1 regulates AKT activity in breast cancer cells [34,35]. As showing in Figure 4A, the expression of Bmi1 and pAKT was both notably higher in SASVO3 cells compared to the SAS-GFP cells. On the other hand, the expression levels of p14 and p21 were reduced in the SASVO3 cells. However, no significant difference was observed for the expression of CD133, CD44, Nanog, and Oct-4 in SAS-GFP and SASVO3 cells (data not shown). Further, to determine whether expression of Bmi1 is correlated with tumorigenicity *in vivo*, we performed a histological examination that detected Bmi1 in the mouse tumor xenografts. Diffuse cytoplasmic Bmi1 staining was detected in the dorsal tissue of the tumor SASVO3 xenografts, but not in dorsal tissue of the SAS-GFP xenografts (Figure 4B).

### Enhanced metastasis in vivo by SASVO3 cells

**Figure 5:**
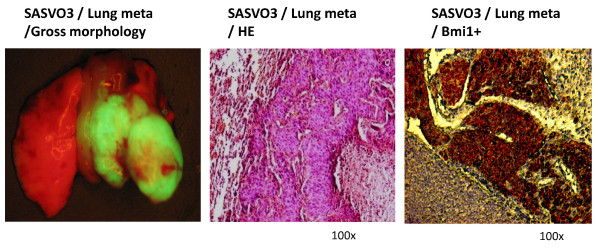
**Pulmonary metastasis generated by SASVO3**. SASVO3 cells were injected into the tail vein and the mice were sacrificed after 90 days to detect lung colonization by tumors. Gross morphology is shown by an illuminating device (*left*). Histological analysis of the lungs for metastatic lesions was performed by H&E staining (*middle*). The expression of Bmi1 in a pulmonary metastatic tumor was detected by IHC (*right*).

As presented earlier, SASVO3 cells display enhanced migration and invasion *in vitro *(see Figure 2), which suggests that SASVO3 may have increased metastatic potential *in vivo*. For epithelial malignancies, the epithelial mesenchymal transition (EMT) is considered to be a crucial event in the metastatic process, and β-catenin is known to be involved in induction of the EMT [36]. To investigate whether SASVO3 cells may have acquired increased metastasis potential, the expression of β-catenin and the ability to induce pulmonary metastasis were examined. As shown in Figure 4A, increased β-catenin expression was detected in lysate from SASVO3 cells, but not in lysate from SAS-GFP cells. Furthermore, pulmonary metastasis was detected in one out of three nude mice that were injected with SASVO3 cells into tail vein (Figure 5, *left*), but none was detected in the three mice injected with SAS-GFP cells. Liver metastasis was also detected in one out of three mice that were injected with SASVO3 cells into the spleen, but none was detected in the three mice injected with SAS-GFP cell (data not shown). Histological examination indicated that there was a significant increase in pulmonary metastatic colonization and angiogenesis in the mice injected with SASVO3 (Figure 5, *middle*). Finally, the pulmonary metastatic tissue displayed strong intranuclear and cytoplasmic Bmi1 staining (Figure 5, *right)*, which is in accordance with the IHC results from the dorsal tumor tissues shown in Figure 4. Together, these results demonstrated that SASVO3 cells displayed increased capability for metastasis *in vivo*.

## Discussion

In this study, we have demonstrated that serial transplantation of a human HNSCC cancer cell line in nude mice can enrich the malignant subpopulation. Furthermore, we provide evidence that the increased malignant potential in the xenograft-derived cells appears to be attributable to the enrichment of a subpopulation that displays an increased expression of Bmi1 and pAKT (Figure 3 and 4), but not CD133, CD44, Nanog, and Oct-4 (unpublished results). The highly tumorigenic cancer stem cells isolated by sphere assay from HNSCC cell lines were previously reported to display an increased expression of CD133, Nanog, and Oct-4 [17]. At present, it is not known why the markers expressed in the subpopulation of serial xenograft-derived SASVO3 differ from the ones expressed in the highly tumorigenic cancer stem cells isolated by sphere assay. Serial passages of cell lines frequently lead to change of characteristics and acquisition of genetic aberrations, resulting in the selection of atypical clones [3,37]. Therefore, the markers on malignant cells of established cell lines may differ from cancer stem cells in original tumors. It is possible that the sphere assay may enrich all of highly malignant cancer stem cells that are present in the established cell lines, while the serial xenotransplantation method may enrich only the original cancer stem cells that produce tumors *in vivo*.

It is interesting to note that the xenograft-derived SASVO3 cells also display increased capability for metastasis (Figure 5), which suggests that the enriched subpopulation may be also highly capable of metastasis. Therefore, the establishing of a highly malignant HNSCC cell line by the xenograft model should be an ideal method for identifying within tumors that subpopulation of cells displaying cancer stem cell properties. Since cancer stem cells have been suspected to underlie the incurable and relapsing nature of many metastatic tumors, our establishment of a highly malignant cell line should pave the road for the future development of new therapies that target these cancer stem cells. In addition, these xenograft-derived cells should be very useful when studying the molecular processes and pathways that are unique to the cancer stem cells.

## Competing interests

The authors declare that they have no competing interests.

## Authors' contributions

CJ performed immunohistochemistry, MT participated in animal practices, CC carried out the rest of research, SC, CH, SL and JL conceived of the study and design research, CC and JL wrote the paper. All authors read and approved the final manuscript.
